# Mitochondrial Elongation and OPA1 Play Crucial Roles during the Stemness Acquisition Process in Pancreatic Ductal Adenocarcinoma

**DOI:** 10.3390/cancers14143432

**Published:** 2022-07-14

**Authors:** Cristian Andres Carmona-Carmona, Elisa Dalla Pozza, Giulia Ambrosini, Barbara Cisterna, Marta Palmieri, Ilaria Decimo, José M. Cuezva, Emanuela Bottani, Ilaria Dando

**Affiliations:** 1 Department of Neurosciences, Biomedicine and Movement Sciences, University of Verona, 37134 Verona, Italy; cristianandres.carmonacarmona@univr.it (C.A.C.-C.); elisa.dallapozza@univr.it (E.D.P.); giulia.ambrosini@univr.it (G.A.); barbara.cisterna@univr.it (B.C.); marta.palmieri@univr.it (M.P.); 2 Section of Pharmacology, Department of Diagnostics and Public Health, University of Verona, 37134 Verona, Italy; ilaria.decimo@univr.it (I.D.); emanuela.bottani@univr.it (E.B.); 3 Departamento de Biología Molecular, Centro de Biología Molecular Severo Ochoa, Consejo Superior de Investigaciones Científicas-Universidad Autónoma de Madrid (CSIC-UAM), Centro de Investigación Biomédica en Red de Enfermedades Raras (CIBERER), ISCIII and Instituto de Investigación Hospital 12 de Octubre, Universidad Autónoma de Madrid, 28049 Madrid, Spain; jmcuezva@cbm.csic.es

**Keywords:** cancer stem cells, pancreatic ductal adenocarcinoma, mitochondrial dynamics, mitochondrial fusion, OPA1

## Abstract

**Simple Summary:**

Pancreatic ductal adenocarcinoma (PDAC) is a highly lethal neoplasia and the currently used treatments are not effective in a wide range of patients. Presently, the evidence points out that cancer stem cells (CSCs) are key players during tumor development, metastasis, chemoresistance, and tumor relapse. The study of the metabolism of CSCs, specifically the mitochondrial alterations, could pave the way to the discovery of new therapeutical targets. In this study, we show that during progressive de-differentiation, pancreatic CSCs undergo changes in mitochondrial mass, dynamics, and function. Interestingly, the silencing of OPA1, a protein involved in mitochondrial fusion, significantly inhibits the formation of CSCs. These results reveal new insight into mitochondria and stemness acquisition that could be useful for the design of novel potential therapies in PDAC.

**Abstract:**

Pancreatic ductal adenocarcinoma (PDAC) is the most common type of pancreatic cancer with an overall 5-year survival rate of less than 9%. The high aggressiveness of PDAC is linked to the presence of a subpopulation of cancer cells with a greater tumorigenic capacity, generically called cancer stem cells (CSCs). CSCs present a heterogeneous metabolic profile that might be supported by an adaptation of mitochondrial function; however, the role of this organelle in the development and maintenance of CSCs remains controversial. To determine the role of mitochondria in CSCs over longer periods, which may reflect more accurately their quiescent state, we studied the mitochondrial physiology in CSCs at short-, medium-, and long-term culture periods. We found that CSCs show a significant increase in mitochondrial mass, more mitochondrial fusion, and higher mRNA expression of genes involved in mitochondrial biogenesis than parental cells. These changes are accompanied by a regulation of the activities of OXPHOS complexes II and IV. Furthermore, the protein OPA1, which is involved in mitochondrial dynamics, is overexpressed in CSCs and modulates the tumorsphere formation. Our findings indicate that CSCs undergo mitochondrial remodeling during the stemness acquisition process, which could be exploited as a promising therapeutic target against pancreatic CSCs.

## 1. Introduction

Pancreatic ductal adenocarcinoma (PDAC) is the most common type of pancreatic cancer and it is the seventh leading cause of cancer-related death in the world with an overall 5-year survival rate of less than 9% [1,2]. The advances in PDAC treatment have been few over the past decades, with adjuvant chemotherapy regimens such as FOLFIRINOX and nab-paclitaxel plus gemcitabine, and surgical resection the only approved therapeutic options. However, these combinations provide only modest improvements in survival rate with considerable toxicity [3]. Furthermore, approximately 80% of PDAC patients are diagnosed at an advanced stage and mostly have no effective treatment options [3,4]. The high aggressiveness of PDAC is associated with increased resistance to conventional therapies, early progression to metastatic disease, and a significant recurrent rate [5]. Recent studies demonstrate that all these aggressive traits are linked to the presence of a subpopulation of cancer cells with a greater tumorigenic capacity, generically named cancer stem cells (CSCs) or tumor-initiating cells [6,7].

CSCs share with normal stem cells the self-renewal and differentiation capacity by asymmetrical division. CSCs are typically in a quiescent state that, under certain stimuli, can proliferate and give rise to the new progeny of tumor cells [8]. CSCs can colonize distant sites from the primary tumor, contributing to metastasis, the leading cause of cancer mortality. Furthermore, the chemoresistance of CSCs to conventional drugs can be attributed to their slow cycle, overexpression of multiple drug transporters, greater DNA repair capacity, and more resistance to mitochondria-mediated cell death than other cells [7,8]. Given that a single CSC could regenerate the whole tumor, the study of CSC hallmarks is crucial for the design of new therapeutic strategies to prevent cancer progression and relapse.

Another important feature of CSCs is their altered metabolism, which remains poorly understood. Some studies have shown that CSCs exhibit increased glycolytic rate and decreased mitochondrial function [9,10], whereas other studies have reported a dependence of these cells on mitochondrial oxidative phosphorylation [11,12], suggesting that CSCs likely present a heterogenous metabolic profile. In fact, our group recently found that gradually de-differentiated CSCs derived from PDAC cell lines shift their metabolism from a glycolytic to an oxidative one, and finally gain a quiescent state. These dormant cells can re-start to proliferate and re-activate the metabolic machinery accumulating lactate, a typical sign of high aggressiveness [13]. This metabolic plasticity of CSCs could be fundamental for their adaptability to different environmental stresses and, eventually, to evade chemotherapy.

Metabolic plasticity would not be possible without mitochondrial remodeling to maintain cellular homeostasis. Mitochondria are not rigid structures, but they are in a constant reorganization in response to metabolic and extracellular insults, a process known as mitochondrial dynamics. In some cells, mitochondria bind together in a connected network, whereas under specific circumstances, mitochondria divide into small fragments [14]. The balance between fusion and fission events is intimately linked to the many functions of mitochondria (e.g., the production of ATP, fatty oxidation, calcium homeostasis, cell signaling, and cell death [14]). The majority of the studies about the metabolic arrangement of CSCs have been performed by culturing cells in vitro for brief periods, with some authors showing that these cells mainly rely on a glycolytic metabolism [15], and others reporting that the inhibition of mitochondrial function in CSCs leads to the loss of stemness by rendering the cells more susceptible to death stimulus [16,17]. Thus, despite these results appear contradictory, they highlight the importance of the role of mitochondria as a promising therapeutic target, showing that these organelles could contribute to preserving a primitive stem-like stage that accounts for CSC chemoresistance.

To determine the role of mitochondria in CSCs cultured for prolonged periods, which may reflect more accurately their quiescent state, we studied the mitochondrial physiology in CSCs at three de-differentiation stages (at 2, 4, and 8 weeks of culture) using a previously characterized PDAC cellular model [13]. Here, we found that CSCs showed a significant increase in mitochondrial mass, enhanced mitochondrial fusion, and a higher mRNA expression of genes involved in mitochondrial biogenesis than the parental cells. These changes are accompanied by a decrease in ATP production, the regulation of OXPHOS complex activity, and reduced assembly into supercomplexes. Furthermore, the silencing of the full form of OPA1, one of the major regulators of mitochondria fusion, shows that CSCs lose some of their stem properties. Altogether, our findings indicate that CSCs undergo a mitochondrial remodeling during the stemness acquisition process, which could be exploited to find vulnerabilities in mitochondrial function as a promising therapeutical target in pancreatic CSCs.

## 2. Materials and Methods

### 2.1. Cell Culture

The pancreatic ductal adenocarcinoma cell line Panc1, here called parental (P) cells, was grown in RPMI-1640 supplemented with 10% FBS and 50 µg/mL gentamicin sulfate (all from Gibco, Life Technologies, Carlsbad, CA, USA), here reported as differentiated-cell medium (DM) and were maintained in standard conditions for a few passages at 37 °C in a 5% CO_2_ atmosphere. CSCs were obtained as described previously [13]. Briefly, adherent cells were washed twice in 1× PBS, trypsinized, centrifuged, washed in 1× PBS, and then cultured in stem-specific medium (SsM) (i.e., DMEM/F-12 without glucose (from Biowest, Nuaillé, France) supplemented with 1 g/L glucose, B27, 1 µg/mL fungizone, 1% penicillin/streptomycin (all from Gibco/Life Technologies, Carlsbad, CA, USA), 5 µg/mL heparin (Sigma/Merck, Darmstadt, Germany), 20 ng/mL fibroblast growth factor (FGF), and 20 ng/mL epidermal growth factor (EGF) (both from PeproTech, London, UK). The cells were cultured in non-treated cell culture flasks ideal for the growth of suspension cells and were maintained at 37 °C with 5% CO_2_ in the SsM until 8 weeks, refreshing twice a week with a new medium. Before each experiment, the cells were passed through a cell strainer (40 µm) to separate and maintain only the cell aggregates/spheres, which were trypsinized to obtain a single-cell suspension. The cell number and cell viability were determined by the trypan blue exclusion test.

### 2.2. RNA Extraction and Real-Time PCR (qPCR)

Total RNA was extracted from 1 × 10^6^ cells using TRIzol Reagent (Life Technologies, Carlsbad, CA, USA) according to the manufacturer’s instructions. RNA integrity was determined by electrophoresis on a denaturing agarose gel. Total RNA (1 µg) was used to synthesize first-strand cDNA by RT-PCR. qPCR was performed in triplicate samples by SYBRGreen detection chemistry using GoTaq qPCR Master Mix (Promega Italia, Milan, Italy) on a QuantStudio™ 3 Real-Time PCR System (Thermo Fisher Scientific, Waltham, MA, USA). The cycling conditions used were: 95 °C for 10 min, 40 cycles at 95 °C for 15 s, 60 °C for 1 min, 95 °C for 15 s, and 60 °C for 15 s. The results were analyzed according to the 2^−∆∆Ct^ method using *SDHA* as an endogenous control. The primers used in this study are listed in Table 1.

### 2.3. mtDNA Quantification

DNA was extracted by digestion with the proteinase K-based protocol. Briefly, cells were resuspended in 700 μL lysis buffer (1 M Tris-HCl, 0.5 M EDTA, 10% SDS, and 5 M NaCl) supplemented with 10 mg/mL of proteinase K (all from Sigma/Merck, Darmstadt, Germany). The samples were incubated at 55 °C for 2 h and then centrifuged for 10 min at 14,000 rpm. DNA was precipitated with 700 μL isopropyl alcohol, centrifuged for 5 min at 14,000 rpm, washed with 70% *v*/*v* ethanol, centrifuged for 5 min at 14,000 rpm, resuspended in 100 μL TE buffer, and quantified with NanoDrop One (Thermo Fisher Scientific, Waltham, MA, USA). Mitochondrial DNA was quantified by SYBRGreen detection chemistry on a QuantStudio™ 3 Real-Time PCR System (Thermo Fisher Scientific, Waltham, MA, USA) according to the 2^−∆∆Ct^ method. The mitochondrial gene MT-ND1 and the nuclear gene B2M were amplified from approximately 20 ng of total DNA (primers are listed in Table 1). The ratio of MT-ND1/B2M equals the mtDNA copy number per cell, providing an indirect measure of the abundance of mitochondria per cell [18]. Digital droplet PCR was performed according to Dolci et al. [19]. Briefly, the amplification reaction was prepared with 0.002 ng of purified DNA, 100 nM of each primer, ddPCR Evagreen Supermix, and 5U/reaction HindIII enzyme (Thermo Fisher Scientific, Waltham, MA, USA). The reaction was then fractionated into ∼20,000 droplets on a QX200 Droplet Generator and end-point PCR was performed. Cycling steps for the ddPCR were as follows: initially, an enzyme activation at 95 °C for 5 min (1 cycle) followed by 40 cycles of denaturation and annealing/extension (each cycle at 95 °C for 30 s and 57 °C for 1 min) and finally signal stabilization (4 °C for 5 min and 90 °C for 5 min, 1 cycle). Droplets were read on a droplet reader and data were analyzed using QuantaSoft™ Software. The fraction of positive droplets was fitted to a Poisson distribution in QuantaSoft™ Software to determine the absolute copy number in units of copies/μL.

**Table 1:** List of human primer sequences used for real-time PCR.

Gene	Primer Pairs–Sequence (5′-3′)	
	Forward	Reverse
NRF1	CCACGTTACAGGGAGGTGAG	TGTAGCTCCCTGCTGCATCT
NRF2	GCGACGGAAAGAGTATGAGC	GTTGGCAGATCCACTGGTTT
TFAM	GTGGTTTTCATCTGTCTTGGC	ACTCCGCCCTATAAGCATCTTG
PGC1α	TGACTGGCGTCATTCAGGAG	CCAGAGCAGCACACTCGAT
CDH1	GACACCAACGATAATCCTCCGA	GGCACCTGACCCTTGTACGT
ZEB1	GTTACCAGGGAGGAGCAGTGAAA	GACAGCAGTGTCTTGTTGTTGTAGAAA
SOX2	GGGAAATGGGAGGGGTGCAAAAGAGG	TTGCGTGAGTGTGGATGGGATTGGTG
NANOG	AGTCCCAAAGGCAAACAACCCACTTC	TGCTGGAGGCTGAGGTATTTCTGTCTC
OCT3/4	GACAGGGGGAGGGGAGGAGCTAGG	CTTCCCTCCAACCAGTTGCCCCAAAC
ND1 *	GTCAACCTCGCTTCCCCACCCT	TCCTGCGAATAGGCTTCCGGCT
B2M *	CGACGGGAGGGTCGGGACAA	GCCCCGCGAAAGAGCGGAAG
DRP1	AAGAACCAACCACAGGCAAC	GTTCACGGCATGACCTTTTT
FIS1	CTTGCTGTGTCCAAGTCCAA	GCTGAAGGACGAATCTCAGG
MFN1	TTGGAGCGGAGACTTAGCAT	TTCGATCAAGTTCCGGATTC
MFN2	AGAGGCATCAGTGAGGTGCT	GCAGAACTTTGTCCCAGAGC
OPA1	GGCCAGCAAGATTAGCTACG	ACAATGTCAGGCACAATCCA

* Primer pairs designed to amplify the genomic DNA (gDNA) taken from [20].

### 2.4. Protein Extraction and Immunoblotting

To prepare the samples, frozen cell pellets were resuspended in lysis buffer (i.e., 1 mM Na_3_VO_4_, 1 mM NaF, 2 mM EDTA, 0.2 mM phenylmethylsulfonyl fluoride (PMSF), 150 mM NaCl, 100× complete protease inhibitor cocktail, and RIPA buffer pH 8.0 (150 mM NaCl, 50 mM Tris-HCl, 1% Igepal, 0.5% sodium deoxycholate, and 0.1% SDS). The lysate was centrifuged at 5000 rpm for 10 min at 4 °C and the supernatant was used for immunoblotting. Protein concentration was measured with the Bradford Reagent (SERVA electrophoresis, Heidelberg, Germany) using bovine serum album as a standard. Next, 30–40 µg protein suspended in SDS loading buffer was run on 12% SDS polyacrylamide gels and electrotransferred to PVDF membranes (Merck Millipore, Burlington, MA, USA). Membranes were then incubated for 1 h at room temperature with blocking solution (i.e., 5% non-fat dried milk in TBST pH 7.5 (100 mM Tris-HCl, 0.1% Tween-20, and 0.9% NaCl)). Then, the membranes were incubated with the primary antibodies at an appropriate dilution in blocking solution overnight at 4 °C. Primary antibodies included: α-Tubulin (1:1500, CP06, Oncogene), total OXPHOS rodent antibody cocktail (1:1000, ab110413, Abcam, Cambridge, UK), anti-human IF1 (1:100, clone14/2) [21], HSP60 (1:2000, Stressgene SPA-807), NDUFA9 (1:1000, #459100, Invitrogen, Waltham, MA, USA), ATP5A (1:100, #459240, Invitrogen), MTCO1 (1:1000, #459600, Novex, Waltham, MA, USA), SDHB (1:1000, #459230, Novex, Waltham, MA, USA), and Core1 (1:1000, #459140, Invitrogen, Waltham, MA, USA). Antibodies against LC3 (1:1500, #2775), DRP1 (1:1000, #8570), P-DRP1 (1:1000, #4494), MFN1 (1:1000, #14739), MFN2 (1:1000, #11925) OPA1 (1:1000, #80471), and TOMM20 (1:1500, #42406) were obtained from Cell Signaling Technology (Danvers, MA, USA). Blots were then incubated with secondary antibodies for 1 h at room temperature and the LiteAblot^®^ Plus substrate (EuroClone, Milan, Italy) was used for the development of immunoreactive bands. The immunocomplexes were visualized by chemiluminescence using the Chemidoc MP imaging system. Densitometric analysis was conducted using ImageJ software (NIH Image, Bethesda, MD, USA).

### 2.5. Transmission Electron Microscopy

Panc1 parental (P) cells and CSCs at 2 weeks (2W), 4 weeks (4W), and 8 weeks (8W) of culture were processed for the ultrastructural morphological and morphometric evaluation of mitochondria using transmission electron microscopy (TEM). Cell samples were fixed with 2.5% glutaraldehyde and 2% paraformaldehyde in 0.1 M phosphate buffer, pH 7.4, at 4 °C for 2 h, then rinsed with PBS, postfixed with 1% OsO4 and 1.5% potassium ferrocyanide for 1 h at 4 °C, dehydrated with acetone, and embedded in Epon 812. Ultrathin sections (70–90 nm thick) were stained with lead citrate for 1 min and observed with a Philips Morgagni transmission electron microscope operating at 80 kV and equipped with a Megaview III camera for digital image acquisition. For morphometric evaluation of the ultrastructural mitochondrial variables (aspect ratio, inner/outer membrane ratio, cristae width, and cristae junction diameter), micrographs of 100 randomly selected mitochondria from 20 different cells were taken for each sample and measurements were performed using ImageJ software (NIH Image, Bethesda, MD, USA). The major axis and minor axis lengths were measured on 50 randomly selected mitochondria (micrographs at ×36,000) for each sample and the aspect ratio parameter was calculated as the ratio of the two lengths. The length of the outer and the inner mitochondrial membrane was measured (micrographs at ×36,000) in 35 mitochondria per sample, and the inner/outer membrane ratio was calculated as an assessment of the cristae extension independent of mitochondrial size. For the cristae width, 15 mitochondria for each sample were examined (micrographs at ×36,000). For the cristae width and junction diameter, micrographs at ×36,000 and ×56,000 were examined, respectively. The means ± SE were calculated for all of the mitochondrial parameters and the statistical comparison was performed with the Kruskal–Wallis non-parametric test followed by the Mann–Whitney test for pairwise comparison. Statistical significance was set at a *p* value ≤ 0.05.

### 2.6. Mitochondria Fluorescent Staining

The mitochondrial structural network was stained using MitoTrackerGreen (Invitrogen, Waltham, MA, USA) according to the manufacturer’s instructions. Briefly, P cells and CSC aggregates were grown in a chambered coverslip for cell live imaging (Ibidi, Grafelfing, Germany), then the medium was removed and the solution containing 50 nM MitoTracker Green was added. Cells were incubated for 30 min in the dark and in standard conditions. After incubation, the staining solution was removed, the cells were washed twice with 1× PBS, and a fresh pre-warmed medium was added. Images were recorded with a confocal laser-scanning fluorescence microscope Leica SP5 (Leica Microsystem, Wetzlar, Germany) at 63× magnification. Subsequently, each 3D stack image was deconvolved using Huygens Professional software package (version 19.04, Scientific Volume Imaging B.V.; Hilversum, The Netherlands, http://svi.nl). The deconvolved images were then processed by Imaris software (version 9.1) (Oxford, UK).

### 2.7. Spheroid Formation Assay

Panc1 parental cells were plated in non-adherent Nunclon Sphera 96-well plates (ThermoFisher Scientific, Waltham, MA, USA) at a density of 300 viable cells per well and grown in SsM. The plate was centrifuged at 1500 rpm for 10 min at room temperature to bring the cells together and then incubated at 37 °C with 5% CO_2_ for 8 days. Images were recorded with an inverted microscope (Axio Vert. A1, Zeiss, Oberkochen, Germany). The spheroid area was measured using ImageJ software (NIH Image, Bethesda, MD, USA).

### 2.8. siRNA Knockdown

Panc1 parental cells were plated in a 6-well plate at a density of 3 × 10^5^ cells per well and grown in standard conditions for 24 h. Then, the cells were transfected with 15 nM of siRNA against OPA1 (SI03019429) and a non-specific negative control (#1027281) purchased from Qiagen (Hilden, Germany) using the Lipofectamine 3000 transfection reagent (Invitrogen, Waltham, MA, USA) according to the manufacturer’s instructions. Briefly, two tubes were prepared as follows: in the first, 3 μL of 15 nM siRNA was added to 125 μL of Opti-MEM (ThermoFisher Scientific, Waltham, MA, USA); in the second, 7 μL of Lipofectamine 3000 was added to 125 μL of Opti-MEM. Both tubes were gently mixed and incubated for 20 min at room temperature, about 250 μL of the mixture was added to the cells. After 48 h, the medium was removed, and cells were washed twice in 1× PBS, trypsinized, centrifuged, washed in 1× PBS, and then cultured in stem-specific medium (SsM) for 10 days to evaluate the expression of stem markers. The knockdown of OPA1 was confirmed using real-time PCR and immunoblotting.

### 2.9. Soft Agar Colony Formation Assay

Panc1 parental cells were transfected as described in Section 2.8. After 48 h, cells were trypsinized and resuspended in 2× RPMI-1640 medium, containing 0.6% agarose (upper layer). The bottom layer of the solidified matrix was made up of 1% agarose in 2× RPMI-1640. Cells were plated in a 6-well plate with a concentration of 5 × 10^4^ cells for each well. All cells were incubated for 35 days. Colonies were photographed by inverted microscope. Diameters of the colonies were measured with ImageJ and the number of colonies composed of more than three cells was counted.

### 2.10. Isolation of Mitochondria

Mitochondria were isolated according to Nuevo-Tapioles et al. [22] with minor modifications. Cell pellets were homogenized in a glass-Teflon homogenizer with a hypotonic buffer (10 mM MOPS, 83 mM sucrose, pH 7.2) and then incubated on ice for 2 min. After incubation, the same volume of hypertonic buffer (30 mM MOPS, 250 mM sucrose, pH 7.2) was added and then centrifuged at 1000× *g* for 5 min at 4 °C to eliminate the nucleus and unbroken cells. The supernatant was centrifuged at 12,000× *g* for 12 min at 4 °C to separate the cytoplasmic fraction. After centrifugation, the pellet was resuspended in buffer A (1 mM EDTA, 10 mM Tris-HCL, 320 mM sucrose, pH 7.4), stored at −80 °C, and used within 6 months.

### 2.11. Cellular ATP Levels

We used a ViaLightTM plus kit (Lonza, Basilea, Switzerland) for the determination of cellular ATP levels. Briefly, Panc1 parental cells and CSCs 2, 4, and 8 weeks were plated in a 12-well plate at a density of 3 × 10^5^ cells per well and grown in DMEM/F-12 without glucose (from Biowest, Nuaillé, France) supplemented with 1 g/L glucose, 10% FBS, 0.365 g/L l-glutamine, and 50 µg/mL gentamicin sulfate for P cells or SsM for CSCs for 24 h. Then, 5 × 10^3^ cells in 100 µL of the medium were transferred to a white 96-well plate in duplicate. ATP was extracted by adding 50 μL of the cell lysis reagent and waiting at least 10 min. The luminescent signal was generated by adding 100 μL of AMR plus and incubating the plate for 2 min at room temperature and recorded with a VictorX Multilabel plate reader (Perkin Elmer, Waltham, MA, USA).

### 2.12. Blue Native Gel Electrophoresis (BNGE)

Samples were prepared according to [23,24]. Briefly, cells were permeabilized in 4 mg/mL digitonin. The resulting mitochondrial enriched fractions were resuspended in 1.5 M aminocaproic acid, 50 mM Bis-Tris/HCl, pH 7, and solubilized in digitonin 4 mg/mg of protein to analyze the supercomplexes. The samples were stored at −80 °C until use. All reagents were purchased from Sigma-Aldrich (Darmstadt, Germany). Blue native gel electrophoresis (BNGE) was performed according to Nijtmans et al. [25] and Wittig et al. [26]. Mitochondrial samples were run on precast native polyacrylamide 3–12% Bis-Tris gels (# BN1001BOX, Invitrogen, Waltham, MA, USA) at 4 °C. Proteins were either transferred on a nitrocellulose membrane (1D-BNGE) or subsequently denatured and run on SDS-PAGE (2D-BNGE). Electroblotting and immunodetection were carried out as described above (Section 2.4).

### 2.13. Mitochondrial Respiratory Complex Activities

Mitochondrial complex activities were measured by spectrophotometric determination as described in [27] with slight modifications. Complex II activity was measured at Abs_60_ using 100 μg of isolated mitochondria in buffer (5 mM MgCl_2_, 25 mM potassium phosphate buffer, 3 mM KCN, and 2.5 mg/mL BSA) containing 30 μM DCPIP, 1 μM antimycin A, 1 μM rotenone, 10 mM succinate, and 6 mM phenazine methosulfate. The activities of complex IV and citrate synthase were measured as described by Spinazzi et al. [28].

### 2.14. Statistical Analysis

Results are presented as mean ± standard error of the mean (SEM) of at least three different biological replicates. Statistical differences were determined by Student’s t-test two-sided and one-way analysis of variance (ANOVA) for multiple comparisons. Data were analyzed using GraphPad Prism software (version 7.0) (San Diego, CA, USA) and statistical significance was defined as *p* < 0.05.

## 3. Results

### 3.1. CSCs Present Increased Mitochondrial Mass

**Figure 1:**
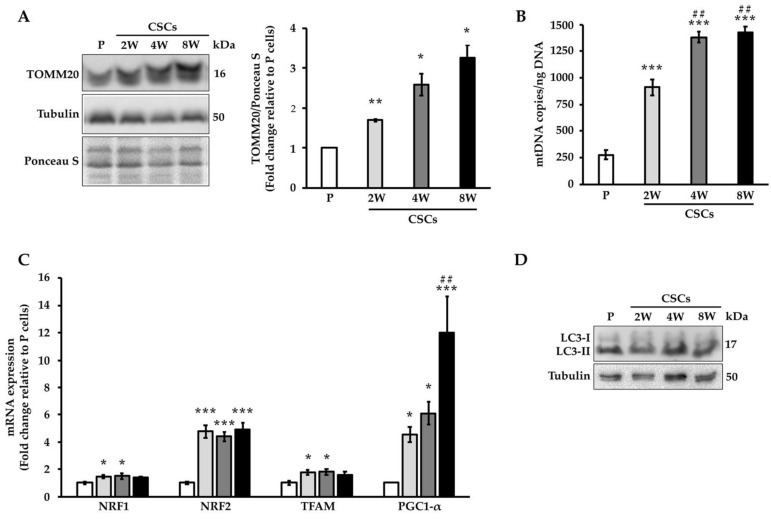
Panc1 cells cultured in the stem-specific medium for 2-, 4-, and 8-weeks exhibited an increase in mitochondrial mass. (**A**) Left panel, representative immunoblot of TOMM20 in Panc1 parental (P) cells and CSCs at 2 weeks (2W), 4 weeks (4W), and 8 weeks (8W) of culture; right panel, densitometry analysis of TOMM20. (**B**) Quantification of mtDNA copies by digital droplet PCR. (**C**) mRNA expression levels of the genes involved in mitochondrial biogenesis in P cells and CSCs. Levels were normalized to *SDHA*. The values are reported as fold change relative to P cells. (**D**) Representative immunoblot of LC3-II. Histogram legends: white: P cells; light gray: CSCs 2 weeks (2W); dark grey: CSCs 4 weeks (4W); black: CSCs 8 weeks (8W). Bars indicate the mean ± SEM of the indicated samples. Statistical legends: Parental vs. CSCs = * *p* < 0.05, ** *p* < 0.01, *** *p* < 0.001; 2W CSCs vs. CSCs = ## *p* < 0.01.

To study the mitochondrial arrangement and function of CSCs, we cultured Panc1 parental (P) cells in a stem-specific medium (SsM) at three different time points, short-term (2 weeks), medium-term (4 weeks), and long-term (8 weeks), as previously described by our group [13]. Indeed, with this cellular model, we showed that gradually de-differentiated CSCs presented metabolic plasticity that ended with the acquisition of a quiescent state [13]. In order to understand the role of mitochondria in the metabolic adaptability of CSCs, we evaluated the protein expression of TOMM20 (Figure 1A), a known mitochondrial outer membrane marker, by Western blotting: the densitometric analysis of the expression of this protein was significantly higher in CSCs relative to the parental cells (Figure 1A). In addition, we also evaluated the expression of another mitochondrial marker (i.e., HSP60), whose expression showed a similar pattern to that of TOMM20 (Supplementary Figure S1A). Furthermore, the quantification of mitochondrial DNA copy numbers (mtDNA) performed through droplet digital PCR also showed a significant increase in mtDNA in CSCs compared to the parental cells, particularly in long-term culture CSCs (at 4 and 8 weeks) (Figure 1B). The quantification of mtDNA was also confirmed by real-time PCR, obtaining similar results (Supplementary Figure S1B). Since an increase in mitochondrial mass might be associated with a higher production of new mitochondria or a reduction in mitochondria turnover through mitophagy, we determined the expression of genes involved in mitochondrial biogenesis and of the autophagosomal marker LC3-II, respectively, in Panc1 CSCs. Figure 1C shows that the mRNA expression levels of four genes regulating mitochondria biogenesis such as NRF1, NRF2, TFAM, and PGC1α, were significantly higher in the CSCs than in the parental cells. Nevertheless, except for PGC1α, for all the other genes, there were no significant differences between CSCs, suggesting that a sustained expression of these genes is necessary to maintain the stemness at all three-time points. On the other hand, the analysis of LC3-II protein expression did not show any marked difference between the parental and CSCs, suggesting that the basal autophagy was similar in both types of cells (Figure 1D). These results indicate that Panc1 CSCs showed more mitochondrial mass, likely attributable to the increased mitochondrial biogenesis.

### 3.2. Mitochondrial Fusion Is Increased in CSCs

**Figure 2:**
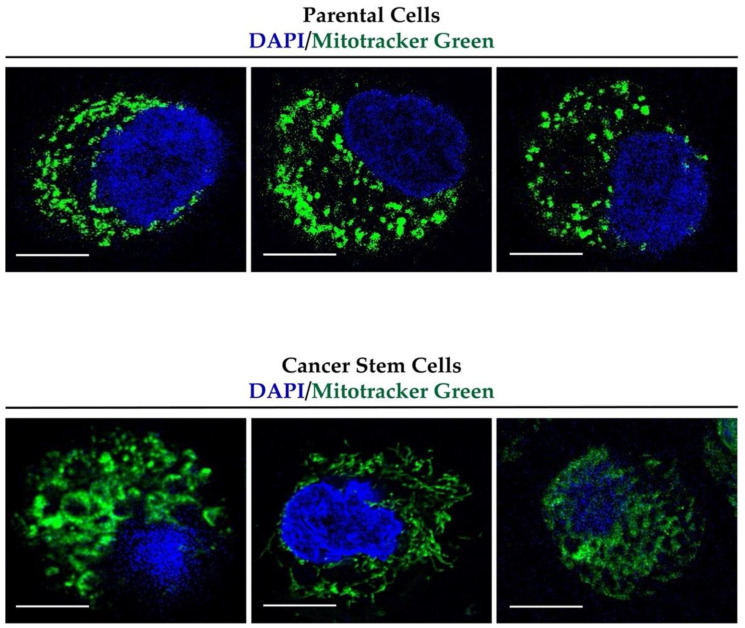
Panc1 CSCs show elongated mitochondria. Representative confocal images of the mitochondria stained with MitoTracker green (green signal) and nucleus stained with DAPI (blue signal) in the parental cells and cancer stem cells at 63× magnification. Scale bar: 10 µm.

To investigate the link between stemness acquisition and mitochondrial dynamics, we compared the mitochondrial morphology of the CSCs with the parental cells using the fluorescent probe MitoTracker Green. This probe is a green-fluorescent dye that localizes to mitochondria regardless of the mitochondrial membrane potential. Figure 2 shows that the mitochondria exhibited a more tubular and less fragmented shape in the CSCs in comparison to the parental cells. Three representative pictures are reported for the P cells and CSCs in Figure 2.

**Figure 3:**
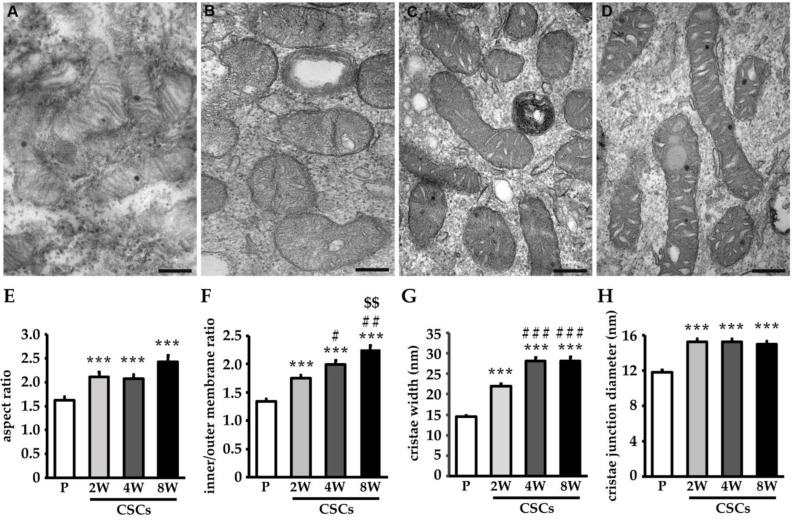
CSCs show alterations of mitochondria structure. Representative transmission electron micrographs of mitochondria of Panc1 parental cells (**A**), and CSCs at 2 weeks (**B**), 4 weeks (**C**), and 8 weeks (**D**) of culture. Scale bar = 200 nm. The histograms represent the means of the aspect ratio (major axis/minor axis length) (**E**), the mitochondrial inner/outer membrane ratio (**F**), the mitochondrial cristae width (**G**), and the mitochondrial cristae junction diameter (**H**). Histogram legends: white: P cells; light gray: CSCs 2 weeks (2W); dark grey: CSCs 4 weeks (4W); black: CSCs 8 weeks (8W). Bars indicate the mean ± SEM of the indicated samples. Statistical legends: Parental vs. CSCs = *** *p* < 0.001; 2W CSCs vs. CSCs = # *p* < 0.05, ## *p* < 0.01, ### *p* < 0.001; 4W CSCs vs. CSCs = $$ *p* < 0.01.

Furthermore, as shown in Figure 3, the TEM images of mitochondria confirmed a more elongated structure of these organelles in CSCs. In fact, the morphometric evaluation of the aspect ratio (Figure 3E), as an index of mitochondrial length, highlighted a significant increase in CSCs at all de-differentiation states. Moreover, the cristae extension significantly increased in CSCs independently of the mitochondrial size, as shown by a higher inner/outer membrane ratio, and this increase was significantly greater at a long culture time point (Figure 3F). The increase in the extent of the cristae in CSCs was associated with a morphological remodeling of the shape of the cristae from typically lamellar in the parental cells (Figure 3A) to a more enlarged one in the de-differentiated cells (Figure 3B–D). As shown by the morphometric evaluation of the mitochondrial cristae width and junction diameter, in CSCs, the cristae were significantly enlarged and this enlargement was particularly evident at 4- and 8-weeks of de-differentiation (Figure 3G). The cristae junction diameter also increased in the CSCs (Figure 3H).

**Figure 4:**
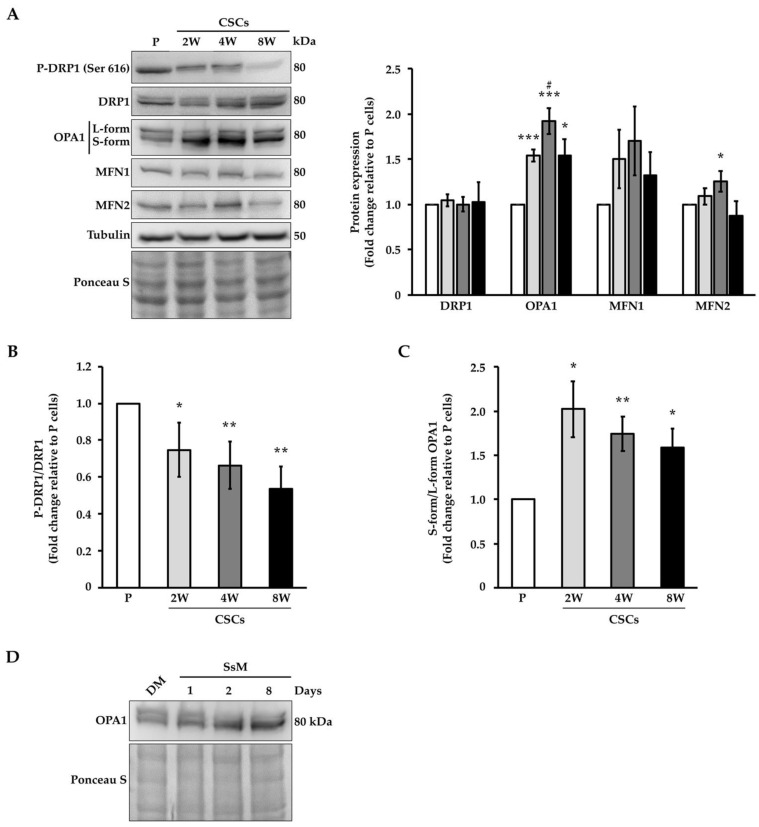
CSC show an increased expression and regulation of fusion markers. (**A**) Left panel, representative immunoblots of the expression of proteins regulating mitochondrial dynamics for parental cells and CSCs; right panel, densitometry analysis average of three different biological samples of the total amount of proteins. Ponceau Stain and tubulin are shown as the loading controls. The ratio of the densitometry analysis of (**B**) phosphorylation of DRP1 (Ser616)/total DRP1 and (**C**) short/long OPA1 isoforms. (**D**) Representative immunoblot of the expression of OPA1 in Panc1 cells grown in differentiated-cell medium (DM) and stem-specific medium (SsM) for 1, 2, and 8 days. Ponceau stain is shown as the loading control. The values are reported as fold change relative to P cells. Histogram legends: white: P cells; light gray: CSCs 2 weeks (2W); dark grey: CSCs 4 weeks (4W); black: CSCs 8 weeks (8W). Bars indicate the mean ± SEM of the indicated samples. Statistical legends: Parental vs. CSCs = * *p* < 0.05, ** *p* < 0.01, *** *p* < 0.001; 2W CSCs vs. CSCs = # *p* < 0.05.

To also confirm the elongation of the mitochondria in CSCs at a molecular level, we evaluated the expression at the mRNA and protein levels of the main mediators of mitochondrial fusion and fission including DRP1, which regulates fission, and OPA-1, MFN-1, and MFN-2, which support fusion. The analysis of the expression at the mRNA levels of these markers showed no significant differences between the CSCs and parental cells independently of the term culture (Supplementary Figure S2). However, it is well-known that mitochondrial dynamics proteins are strongly regulated at the post-translational level (e.g., the phosphorylation of DRP1 on Ser616 enhances its function supporting mitochondrial fission). Thus, we quantified the protein expression levels of these fission and fusion markers by immunoblot, as shown in Figure 4A. Our data showed that CSCs exhibited significantly lower phosphorylation of DRP1 at Ser616 than the parental cells and this decrease was substantial in the CSCs in the medium- and long-term culture (i.e., 4- and 8-weeks (Figure 4B)). On the other hand, the expression of OPA1 was significantly higher in the CSCs in comparison to the parental cells (Figure 4A), whereas two other fusion markers (MFN1 and MFN2) showed a similar trend of increase only after 4 weeks of culture. Interestingly, the quantitative analysis of OPA1 by Western blot (Figure 4C) revealed that CSCs presented an imbalance in the isoforms of OPA1, predominating short isoforms. In addition, as shown in Figure 4D, the transition from long (upper band) to short isoform (lower band) takes place during the first days of culture in SsM, which suggests that the cleavage of OPA1 is an early event and might be important during the process of stemness acquisition. In addition to OPA1, the expression of MFN2 is increased in CSCs at 4 weeks of culture. Taken together, these data indicate that Panc1 CSCs showed increased mitochondrial fusion; note, the higher expression of short isoforms of OPA1 in the CSCs than in the parental cells.

### 3.3. OPA1 Modulates the Tumorsphere Formation

**Figure 5:**
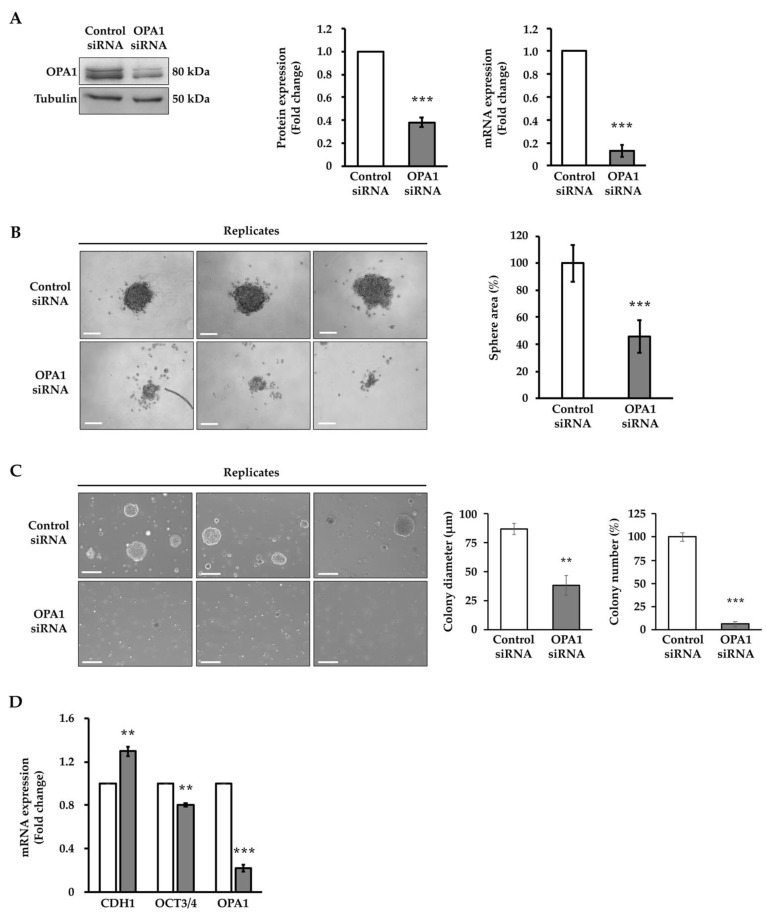
Knockdown of OPA1 reduces the sphere formation in Panc1 cells. (**A**) Protein and mRNA expression levels of OPA1 after its silencing in Panc1 parental cells: the left panel depicts a representative immunoblot against OPA1 in Panc1 cells after 72 h of transfection using OPA1 siRNA. The right panel shows the mRNA expression levels of OPA1 in Panc1 cells after 48 h of transfection using OPA1 siRNA. (**B**) After 48 of transfection with OPA1 siRNA, Panc1 cells were seeded in non-adherent Nunclon Sphera 96-well plates for 8 days. The left panel is the bright field microscopy image of the spheres in the control and silenced cells and the right panel is the quantification of the sphere size. (**C**) Representative images of the soft agar colony formation. After 48 h of transfection with OPA1 siRNA, Panc1 cells were seeded for soft agar colony assay and bright field microscopy images were taken after 35 days of culture. Scale bar = 100 μm. The diameter of the colonies generated by the OPA1 silenced cells and control cells was measured with ImageJ and reported in μM. The number of the colonies composed of more than three cells is reported as fold change relative to the control cells. (**D**) qPCR analysis of the relative mRNA expression levels of the key EMT/stemness genes (*CDH1* and *OCT3/4*) in Panc1 cells transfected with siRNA OPA1 and grown in the stem-specific medium for 10 days. OPA1 mRNA expression was also quantified. Levels were normalized to *SDHA*. Histogram legends: white: control siRNA cells; grey: OPA1 siRNA cells. The values are reported as fold change relative to the control siRNA cells. Statistical legends: control siRNA cells vs. OPA1 siRNA cells = ** *p* < 0.01, *** *p* < 0.001.

Considering the overexpression of OPA1 in CSCs, next, we studied the role of this protein in the determination of the stem features of CSCs by silencing OPA1 with siRNA. Real-time PCR and immunoblotting were used to compare the expression of OPA1 in the resulting cells with their corresponding controls (Figure 5A). Roughly, the reduction in OPA1 was 80% and 60% at the mRNA and protein levels, respectively. To study the role of OPA1 in the determination of stemness, first, we analyzed the tumorsphere forming ability: we seeded transfected cells in SsM and measured the sphere size after 8 days of incubation in standard conditions (i.e., cells cultured in the medium of differentiated cells). Figure 5B shows that the sphere size was significantly reduced to more than 50% in the siOPA1 cells compared with the control cells. To further investigate the effect of OPA1 silencing on stemness, we analyzed the ability of the cells to form colonies through soft agar after OPA1 silencing. Indeed, Figure 5C shows that the depletion of OPA1 drastically decreased the capability of the cells to generate colonies that presented a significantly smaller diameter in comparison to the control cells, further supporting the key role of this protein in the determination of undifferentiated features of the cells. It is worthwhile considering that the cell viability after the knockdown of OPA1 was similar to that of the control cells (data not shown). Finally, we also assessed the expression of some key stem markers using real-time PCR (Figure 5D). *OCT3/4*, which is a known marker of stemness [13], showed a significant decrease in its expression in the siOPA1 cells compared with the control cells. Accordingly, the expression of *CDH1*, one of the main mediators of epithelial-to-mesenchymal transition (EMT) that we previously showed to be decreased in CSCs [13], was significantly increased in the siOPA1 cells relative to the control cells. The expression of the other stem markers remained unaltered after OPA1 silencing (Supplementary Figure S3). Interestingly, the levels of OPA1 mRNA remained successfully decreased in the Panc1 CSCs after 10 days of incubation in SsM using the siRNA method (Figure 4D). These results indicate that OPA1 modulates the formation of tumorspheres, likely through a possible role in EMT.

### 3.4. CSCs Show Modulation of Mitochondrial Respiratory Complex Amounts, Activity, and Assembly into Supercomplexes

**Figure 6:**
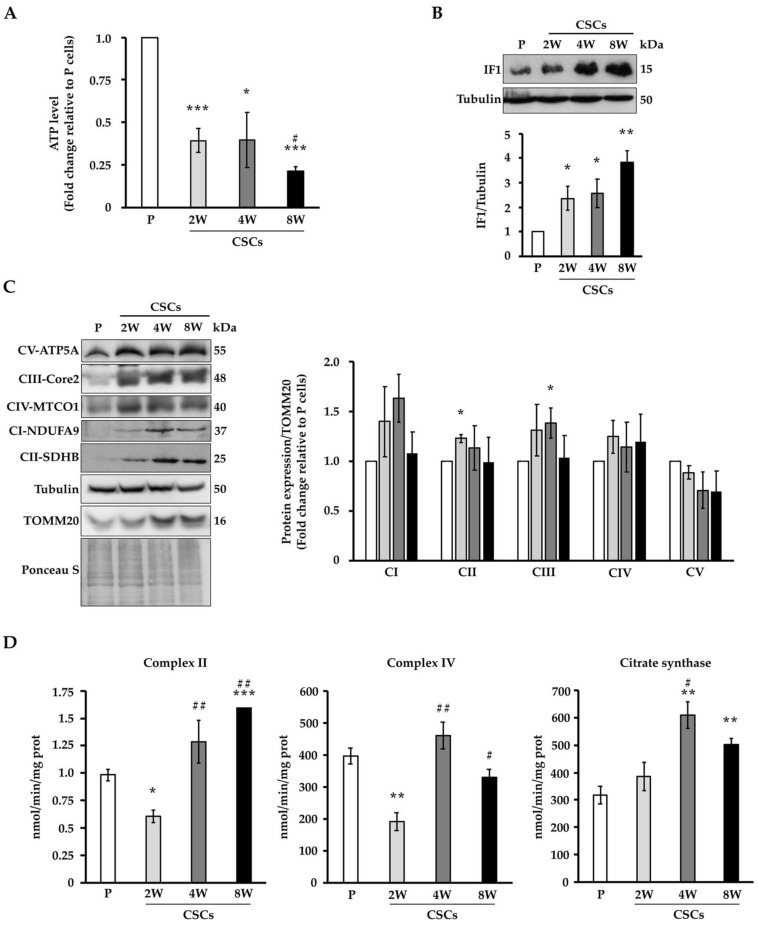
CSCs show modulation of the mitochondrial respiratory complex amounts and activities. (**A**) ATP levels of Panc1 parental and CSCs. (**B**) Representative immunoblot of ATPase inhibitory factor 1 (IF1) and densitometric quantification (histogram) of three different biological replicates of IF1 expression. Tubulin is shown as a loading control. (**C**) Right panel, representative immunoblots of three different biological samples of the expression of mitochondrial respiratory proteins from complex I (NDUFA9), complex II (SDHB), complex III (core2), complex IV (MTCO1), and complex V (ATP5A). Ponceau Stain, tubulin, and TOMM20 are shown as loading controls. Left panel, densitometric quantification of mitochondrial respiratory proteins normalized to TOMM20. (**D**) The histograms show the enzymatic activity of the complex II, complex IV, and citrate synthase of three different biological replicates by spectrophotometry. The values are reported as fold change relative to P cells. Histogram legends: white: P cells; light gray: CSCs 2 weeks; dark grey: CSCs 4 weeks; black: CSCs 8 weeks. Bars indicate the mean ± SEM of different experiments as indicated. Statistical legends: Parental vs. CSCs = * *p* < 0.05, ** *p* < 0.01, *** *p* < 0.001; 2W CSCs vs. CSCs = # *p* < 0.05, ## *p* < 0.01.

In order to evaluate the energetic requirements of P cells and CSCs, we analyzed the global ATP production (Figure 6A). Our data showed that CSCs significantly dropped their ATP content, and CSCs at 8 weeks of culture showed the lowest level, in agreement with their prominent quiescent state [13]. Since the main source of ATP production resides in mitochondria, we investigated their functionality, focusing on the expression of proteins involved in the electron transport chain (ETC) that have been recently proposed as possible therapeutical targets in refractory cancers such as PDAC [29,30]. In line with the previous observations, the total expression levels of the ATPase inhibitor factor (IF1), a physiological inhibitor of mitochondrial ATP synthase, were significantly increased in the CSCs compared to the parental cells, notably in CSCs at 8 weeks (Figure 6B). On the other hand, the immunoblotting (Figure 6C) and the relative densitometric analysis (Supplementary Figure S4) of some subunits of the OXPHOS complexes showed a significant increase in the proteins NDUFA9 (CI), SDHB (CII), COREII (CIII), MT-COI (CIV), and ATP5A (CV) in the CSCs relative to the parental cells, suggesting an increase in the ETC proteins *per* cell. However, when normalized to the mitochondrial marker TOMM20, the expression of the OXPHOS subunits remained mostly unchanged, suggesting that mitochondria did not increase their ETC units (Figure 6C right panel). In order to analyze whether OXPHOS complexes in CSCs had altered functionality, we investigated the activity of the respiratory complexes II and IV by spectrophotometric assays (Figure 6D). A significantly decreased activity was observed for both complexes in the CSCs at 2 weeks compared to the parental cells and other CSCs. At the next step of de-differentiation (i.e., CSCs at 4 weeks), there was an increase in these activities, whereas CSCs at 8 weeks of culture showed a trend of decrease in the CIV in comparison to the previous time point. Moreover, the activity of the TCA cycle enzyme citrate synthase was strongly increased at 4 weeks and at 8 weeks of culture. Altogether, these data indicate that, despite the increase in mitochondrial mass *per* cell in the CSCs, the expression of the ETC complexes was not increased in comparison to the parental cells, whereas there was a modulation of the catalytic activity of complexes II and IV.

**Figure 7:**
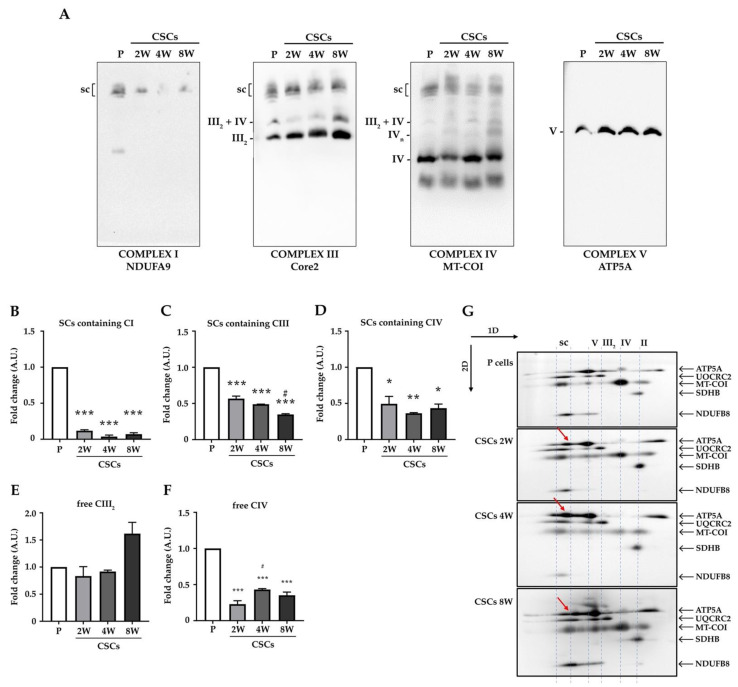
CSCs show a decreased amount of mitochondrial respiratory supercomplexes (SCs) at long culture term. (**A**) 1D-BNGE analysis of mitochondrial supercomplexes (SC) in the Panc1 parental and CSCs. Representative immunoblots of OXPHOS complexes and SC detected with the antibodies indicated below each blot. The migrations of SC, III2 + IV, III_2_, IVn, IV, and V are shown. (**B**–**D**) Quantifications of complex I- (**B**), III- (**C**), IV-containing SC (**D**), free III_2_ (**E**), and IV (**F**) were performed through normalization on each signal to the amount of CV and expressed as fold change compared to the parental cells. Error bars indicate the mean ± SEM. Statistical analysis was performed with one way ANOVA and Tukey’s multiple comparisons test: Parental vs. CSCs = * *p* < 0.05, ** *p* < 0.01, *** *p* < 0.001; 2W CSCs vs. CSCs = # *p* < 0.05. (**G**) 2D-BNGE analysis on parental and CSCs. Red arrows indicate the appearance of CV dimers in CSCs that are not present in the parental cells.

Because changes in mitochondrial OXPHOS efficiency are also linked to alteration in the formation of respiratory supercomplexes [31], we further investigated the assembly of ETC complexes in parental cells and CSCs through Blue Native Gel Electrophoresis (BNGE). Since complex V does not participate in supercomplexes and did not change in our experimental settings (Figure 6C), we compared the relative amount of respiratory supercomplexes between the parental and CSCs’ previous normalization to CV. Interestingly, we found a substantial reduction in CI-, CIII-, and CIV-containing supercomplexes in CSCs (Figure 7A–D), which was paralleled by an increase in free CIII_2_ (Figure 7E) and a reduction in free CIV_2_ (Figure 7F). Similar results were obtained after normalization to the CII signal (not shown). Furthermore, 2D-BNGE showed that complex V, which is visible only in a monomeric state in parental cells, was also present in a dimeric state in CSCs (Figure 7G, red arrows). These data indicate that a substantial reorganization of ETC units occurs during the de-differentiation process of CSCs.

## 4. Discussion

Cancer stem cells (CSCs) are considered as primarily responsible for the aggressiveness of pancreatic ductal adenocarcinoma and relapse after chemotherapy [32]. Thus, the identification of the main features of these cells as possible therapeutical targets is crucial to eliminate the tumor by the root. Considering that CSCs possess different grades of differentiation and metabolic profiles within the tumor, some authors have proposed the mitochondrion as a key player in metabolic plasticity and drug resistance profile [33,34]. With this idea in mind, numerous studies have been performed to elucidate the role of mitochondria in cancer metabolism, however, the precise mechanisms underlying the alterations of mitochondria in CSCs remain controversial [11,12,35]. One of the main issues is the method of isolation or obtainment of CSCs. Indeed, the isolation of CSCs from primary tumors based on the expression of stem markers represents a big challenge because these cells quickly return to the heterogeneity present in the tumor within a few days of culture [11] and, more importantly, the use of specific surface markers would select only a subpopulation of CSCs that is not always representative of the whole tumor heterogeneity [36,37]. This has led other authors to study the characteristics of CSCs on short-term culture; however, it has been shown that this stem model does not necessarily reflect the metabolic plasticity and quiescence-like state associated with CSC phenotypes [11]. For these reasons, we decided to study the mitochondrial function in an in vitro model of CSCs derived from the Panc1 cell line cultured in a stem-specific medium (SsM) at three different time points, short-term (2 weeks), medium-term (4 weeks), and long-term (8 weeks) [13]. In this model, CSCs at three-time points showed a significantly high expression of stem and EMT markers, although long-term culture cells showed the highest grade of stemness. During the process of de-differentiation, these cells switch from a glycolytic to an oxidative metabolism to finally reduce their metabolism to gain a quiescent state [13]. In the present work, we showed that Panc1 CSCs, regardless of the term culture, increased the mitochondrial mass and the expression of genes involved in mitochondrial biogenesis in comparison to the parental cells. These findings are consistent with prior studies where CSCs from different tumor types exhibited enhanced mitochondrial mass [38,39,40]. An increase in mitochondrial biogenesis is commonly associated with a higher tumorigenic rate, metastatic potential [41,42], and evasion of chemotherapy [40]. Given the increased mitochondrial mass in CSCs, we focused our attention on the morphology of these organelles and on the expression of proteins involved in their dynamics [14], with special attention to the protein OPA1, a key player in this scenario. Concerning mitochondria dynamics, our data showed that CSCs expressed higher levels of OPA1, a protein that determines mitochondria fusion, together with lower levels of DRP1 phosphorylation on Ser616, a post-translation modification that inhibits the function of this mitochondrial fission protein, in comparison to the parental cells. Interestingly, the electrophoretic profile of OPA1 revealed an increase in S-isoforms and a decrease in L-isoforms in CSCs in comparison to the parental cells, and was more evident in the CSCs at 2 and 4 weeks. These S-isoforms have been shown to support a more efficient energetics preservation and to regulate the metabolic shift from glycolysis to mitochondrial respiration in human fibroblasts [43,44]. In addition, OPA1 knockdown reduces the tumorsphere size and alters the expression of OCT3/4 and CDH1, key stem and EMT markers. Despite the effect of OPA1 silencing on stemness appearing to be crucial, other authors have reported that CSCs exhibited highly fragmented mitochondria mediated by DRP1 activation [16,45,46]. These differences could be due to the fact that in these studies, the CSCs were grown in different conditions and for a short period of culture. In fact, our findings call into question the effectiveness of using DRP1 inhibitors as therapeutic agents to eliminate pancreatic CSCs [46], considering that during the process of stemness acquisition, these cells could present an oxidative phenotype [11,12,13] that is favored by mitochondrial fusion [47]. In contrast, our results led us to propose the inhibition of OPA1 as a possible therapeutical target to reduce the mitochondrial fusion in CSCs, and eventually disturb their metabolism. Indeed, some authors have recently shown the crucial role of OPA1 in tumor cell metabolism [48] and angiogenesis [49]. Concerning the alterations of mitochondria morphology, despite the previous assumption about the role of L-/S-OPA1 oligomers in the determination of cristae structure, it has been interestingly shown that S-OPA1 alone is fundamental to determine a normal mitochondrial cristae structure [43]. On the other hand, our data showed that CSCs possess elongated mitochondria with an increased cristae width and junction diameter, together with an increased expression level of the short form of OPA1 (S-OPA1) in comparison to the parental cells. Although these data are apparently in contrast to the literature, future studies are necessary to define the distribution of the two forms of OPA1 in the mitochondria and to analyze the effect of OPA1 silencing on the determination of the cristae structure in CSCs. Concerning the functionality of mitochondria characterized by increased cristae width, a study performed on HACD1-KO mice showed that widened cristae presented a decreased content of anionic lipids with an alteration in the cristae shape, a reduction in proton translocation efficiency, and impaired ATP production [43]. These observations may pave the way for future investigations on the connection of S-OPA1 accumulation, the alteration in ETC functionality, and assembly of supercomplexes in CSCs. Although it has been reported that CSCs present metabolic plasticity, relying mainly on mitochondrial OXPHOS for energy production, the role of mitochondrial respiratory complexes and their assembly in this metabolic adaptation remain unexplored. Our study shows that the expression of the ETC complexes I–V at protein levels remained unaltered in CSCs, considering the higher mitochondrial mass. On the other hand, the activities of complex II and IV were modulated based on the specific metabolic features of CSCs, as we previously reported [13]. Indeed, we recently showed that during progressive de-differentiation, CSCs undergo metabolic plasticity by first preferring glycolysis, then oxidative phosphorylation, and finally gaining the quiescent state. In line with this, CSCs at 2 weeks showed the lowest enzymatic activity of complexes II and IV, supporting that short-term CSCs prefer a glycolytic metabolism. Consistently, the increased activity of these two complexes in CSCs at 4 weeks and the decrease in complex IV activity at 8 weeks of culture further corroborate our previous findings in which we showed that, at medium-term culture, the CSCs increased their oxidative capacity, instead of at a longer culture time, where they decreased the metabolic requests and entered into a quiescent state [13]. However, the mild modulations in CII and CIV activities were not proportional to the massive increase in the mitochondrial mass and CS activity that characterizes the CSCs. Accordingly, the global ATP content significantly dropped in CSCs; possibly due to the increased expression of IF1. Moreover, the respiratory supercomplexes, which have been proposed to optimize the electron transport chain activity [50] were significantly decreased in the CSCs compared to the parental cells.

In addition, after mitochondrial solubilization, we observed the presence of ATP synthase dimers in CSCs, which is favored by the expression of mitochondrial ATPase inhibitory factor 1 (IF1) in cells [51] and in mouse tissues in vivo [52,53]. It has been shown that mitochondrial respiration and the oligomycin-sensitive respiration, which provides a measure of the ATP synthetic activity of the enzyme, are significantly reduced as the dose of IF1 increases in the cells [52,53]. In addition, the expression of IF1 was significatively increased in CSCs relative to parental cells, particularly in CSCs at 8 weeks. The overexpression of this factor in CSCs at 8 weeks could explain in part the low ATP-linked respiration reported in these cells by Ambrosini et al. [13] and the low levels of total ATP production. Further observations are needed to explain the role of IF1 during the process of de-differentiation and in the maintenance of the cancer stem-like stage.

Altogether, our results show that the CSCs presented altered mitochondrial arrangement and functionality, together with the previously described alterations of the energetic metabolism [13]. From a therapeutical point of view, these data could be exploited in the future to design therapies that directly affect cancer cells to avoid their entrance into the quiescent state, a hallmark of aggressiveness and relapse. A therapeutic option may be represented by a direct effect on mitochondria elongation and, in particular, OPA1, whose depletion has been shown to decrease the stem features of CSCs.

## 5. Conclusions

In this study, we showed that pancreatic CSCs presented increased mitochondrial mass and elongated morphology. Despite this enhanced mitochondrial mass, the global functionality of these organelles in CSCs was decreased, opening the way to future investigations regarding the connection of mitochondria fusion and the entrance into the quiescent state, a typical characteristic of CSCs. Interestingly, we demonstrated a key role for OPA1, a protein that drives mitochondria fusion, in the determination of the stem features of the cells. Altogether, our results add new insights into the exploitation of mitochondrial features to design novel potential therapies against PDAC CSCs.

## Data Availability

Not applicable.

## Supplementary Materials

The following supporting information can be downloaded at: www.mdpi.com/article/10.3390/cancers14143432/s1, Figure S1: Panc1 cells cultured in the stem-specific medium exhibited an increase in the mitochondrial mass; Figure S2: The expression of genes involved in mitochondrial dynamics in Panc1 cells cultured in the stem-specific medium for 2-, 4-, and 8-weeks; Figure S3: qPCR analysis of relative mRNA expression levels of the key EMT/stemness genes in Panc1 cells transfected with OPA1 siRNA and grown in the stem-specific medium for 10 days; Figure S4: CSCs show the modulation of mitochondrial respiratory complex expression normalized on tubulin.
