# Supplementary material for: Mitochondrial Elongation and OPA1 Play Crucial Roles during the Stemness Acquisition Process in Pancreatic Ductal Adenocarcinoma
Source: Cancers (Basel). 2022 Jul 14;14(14):3432. doi: 10.3390/cancers14143432 (PMC9322438; doi:10.3390/cancers14143432)
Supplement: Supplementary file 1 [file cancers-14-03432-s001.zip › cancers-1773998-supplementary/Full_length_blots_16_July_2022.pdf]

Mitochondrial elongation and OPA1 play crucial roles during the stemness acquisition process in pancreatic ductal adenocarcinoma

Cristian Andres Carmona-Carmona[1], Elisa Dalla Pozza[1], Giulia Ambrosini[1], Barbara Cisterna[1], Marta Palmieri[1], Ilaria Decimo[2], José M. Cuezva[3], Emanuela Bottani[2] and Ilaria Dando[1,*]

Afifiliations

1. Department of Neurosciences, Biomedicine and Movement Sciences, University of Verona, 37134 Verona, Italy; cristianandres.carmonacarmona@univr.it (C.A.C.-C.); elisa.dallapozza@univr.it (E.D.P.); giulia.ambrosini@univr.it (G.A.); barbara.cisterna@univr.it (B.C.); marta.palmieri@univr.it (M.P.).

2. Section of Pharmacology, Department of Diagnostics and Public Health, University of Verona, 37134 Verona, Italy; ilaria.decimo@univr.it (I.D.); emanuela.bottani@univr.it (E.B.).

3. Departamento de Biología Molecular, Centro de Biología Molecular, Consejo Superior de Investigaciones Científicas Universidad Autónoma de Madrid (CSIC-UAM), Centro de Investigación Biomédica en Red de Enfermedades Raras (CIBERER), ISCIII and Instituto de Investigación Hospital 12 de Octubre, Universidad Autónoma de Madrid, 28049 Madrid, Spain; jmcuezva@cbm.csic.es (JMC).

Correspondence to:

Prof Ilaria Dando; Department of Neurosciences, Biomedicine and Movement Sciences, University of Verona, 37134 Verona, Italy. Email: ilaria.dando@univr.it; Tel.: +39-045-8027169

Supplementary full-length blots:

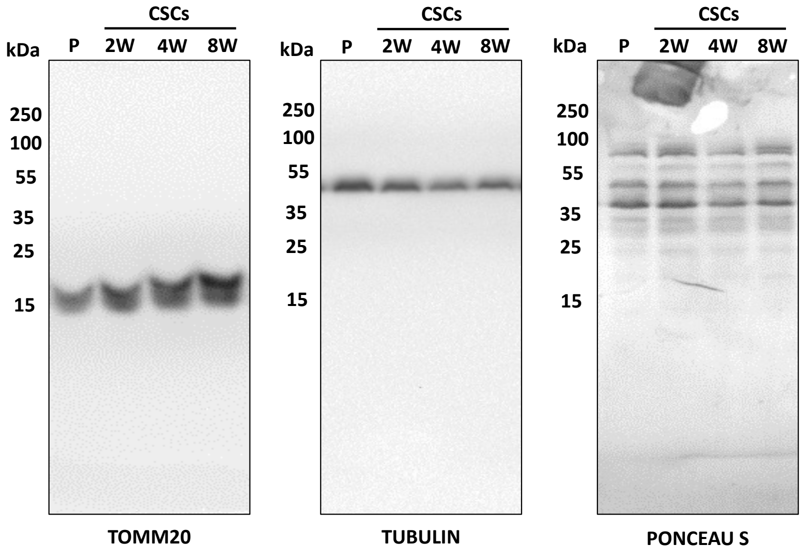

Supplementary blot 1. Full-length western blot from figure 1A. Representative immunoblot of TOMM20 and loading control (Tubulin and Ponceau S) in Panc1 parental (P) cells and CSCs at 2 weeks (2W), 4 weeks (4W), and 8 weeks (8W) of culture. 17% SDS-PAGE gel.

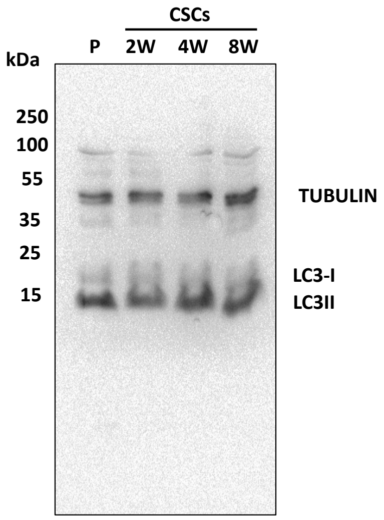

Supplementary blot 2. Full-length western blot from figure 1D. Representative immunoblot of LC3 and loading control (Tubulin) in Panc1 parental (P) cells and CSCs at 2 weeks (2W), 4 weeks (4W), and 8 weeks (8W) of culture. 17% SDS-PAGE gel.

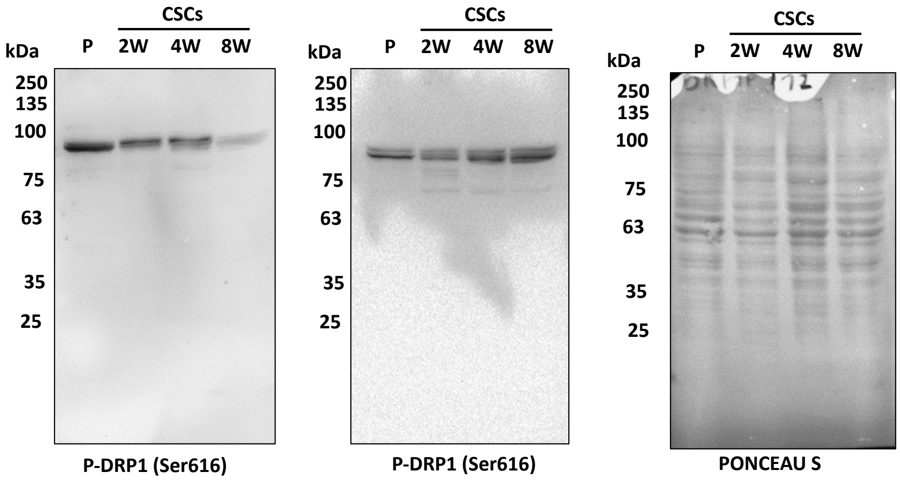

Supplementary blot 3. Full-length western blot from figure 4A. Representative immunoblot of P-DRP1, DRP1, and loading control (Ponceau S) in Panc1 parental (P) cells and CSCs at 2 weeks (2W), 4 weeks (4W), and 8 weeks (8W) of culture. 12% SDS-PAGE gel

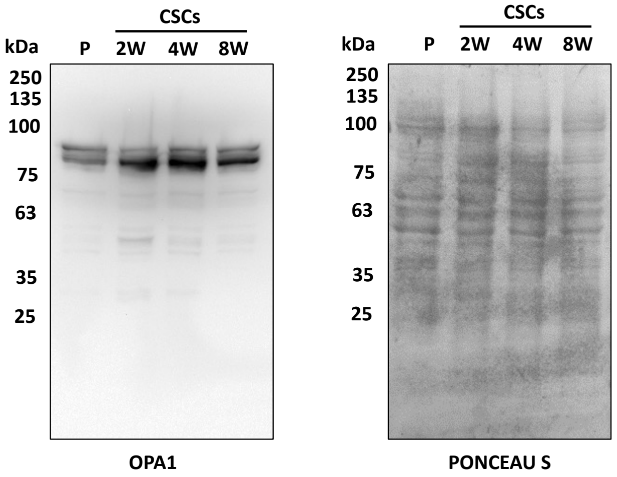

Supplementary blot 4. Full-length western blot from figure 4A. Representative immunoblot of OPA1 and loading control (Ponceau S) in Panc1 parental (P) cells and CSCs at 2 weeks (2W), 4 weeks (4W), and 8 weeks (8W) of culture. 12% SDS-PAGE gel.

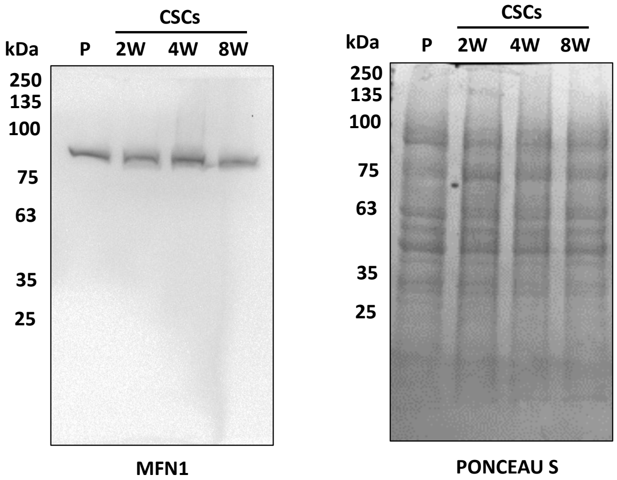

Supplementary blot 5. Full-length western blot from figure 4A. Representative immunoblot of MFN1 and loading control (Ponceau S) in Panc1 parental (P) cells and CSCs at 2 weeks (2W), 4 weeks (4W), and 8 weeks (8W) of culture. 12% SDS-PAGE gel.

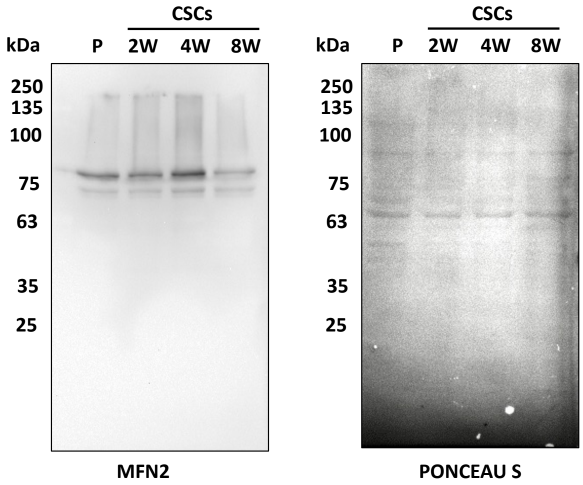

Supplementary blot 6. Full-length western blot from figure 4A. Representative immunoblot of MFN2 and loading control (Ponceau S) in Panc1 parental (P) cells and CSCs at 2 weeks (2W), 4 weeks (4W), and 8 weeks (8W) of culture. 12% SDS-PAGE gel.

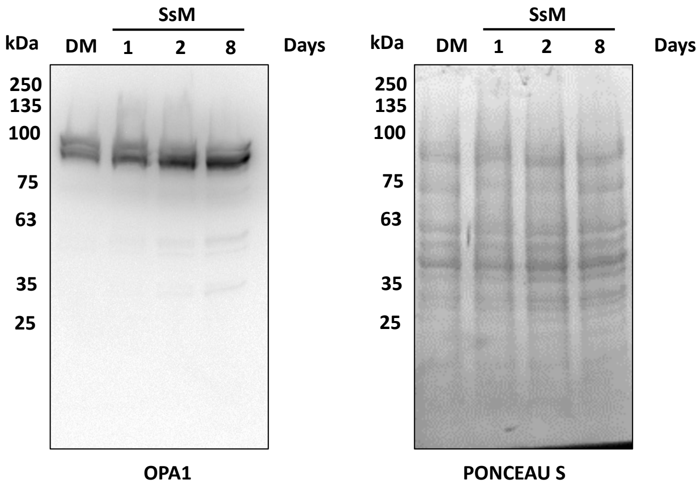

Supplementary blot 7. Full-length western blot from figure 4D. Representative immunoblot of the expression of OPA1 in Panc1 cells grown in differentiated-cell medium (DM) and stem-specific medium (SsM) for 1, 2, and 8 days. Ponceau stain is shown as the loading control**.** 12% SDS-PAGE gel.

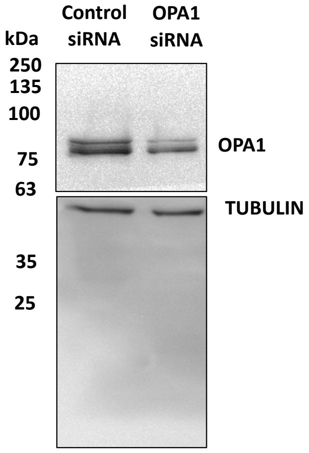

Supplementary blot 8. Full-length western blot from figure 5A. Representative immunoblot against OPA1 in Panc1 cells after 72 hours of transfection using OPA1 siRNA.

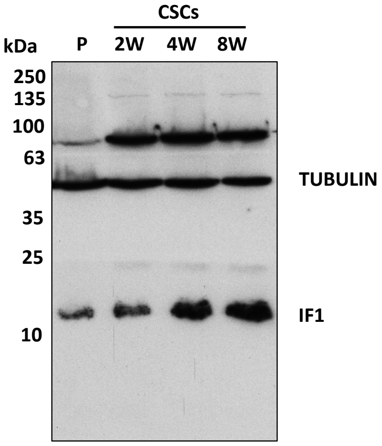

Supplementary blot 9. Full-length western blot from figure 6B. Representative immunoblot of IF1 and loading control (Tubulin) in Panc1 parental (P) cells and CSCs at 2 weeks (2W), 4 weeks (4W), and 8 weeks (8W) of culture. 12% SDS-PAGE gel.

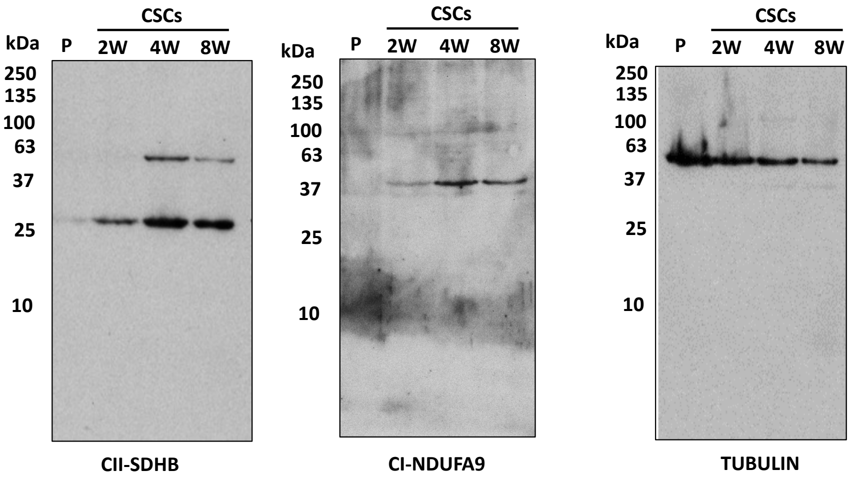

Supplementary blot 10. Full-length western blot from figure 6C. Representative immunoblot of SDHB, NDUFA9, and loading control (Tubulin) in Panc1 parental (P) cells and CSCs at 2 weeks (2W), 4 weeks (4W), and 8 weeks (8W) of culture. 12% SDS-PAGE gel.

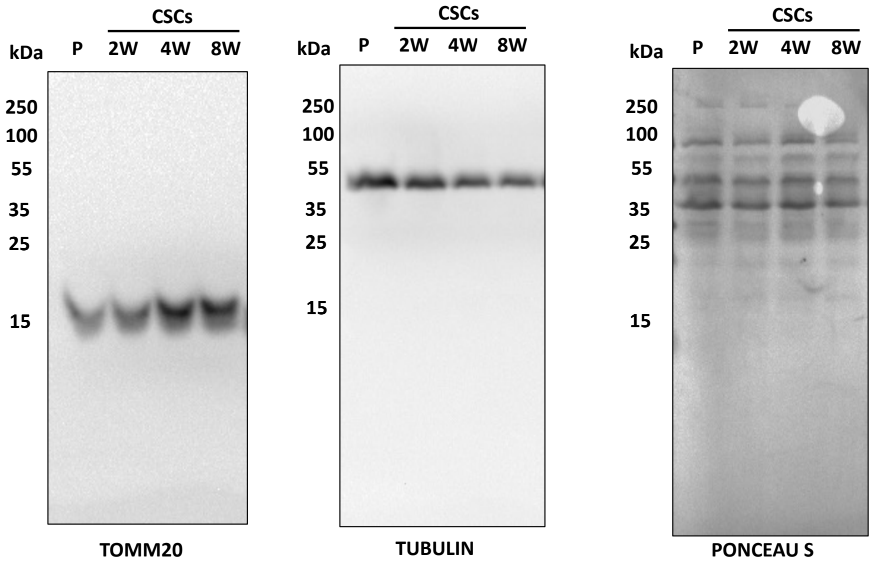

Supplementary blot 11. Full-length western blot from figure 6C. Representative immunoblot of TOMM20 and loading control (Tubulin and Ponceau S) in Panc1 parental (P) cells and CSCs at 2 weeks (2W), 4 weeks (4W), and 8 weeks (8W) of culture. 17% SDS-PAGE gel.

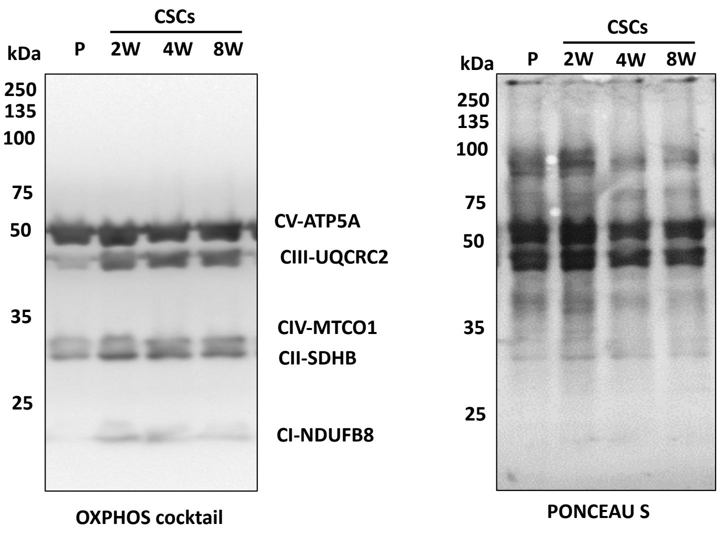

Supplementary blot 12. Full-length western blot from figure 6C. Representative immunoblot of NDUFB8 (Complex I), SDHB (Complex II), UQCRC2 (Complex III), MTCO1 (Complex IV), and ATP5A (Complex V), and loading control (Ponceau S) in Panc1 parental (P) cells and CSCs at 2 weeks (2W), 4 weeks (4W), and 8 weeks (8W) of culture. 12% SDS-PAGE gel.

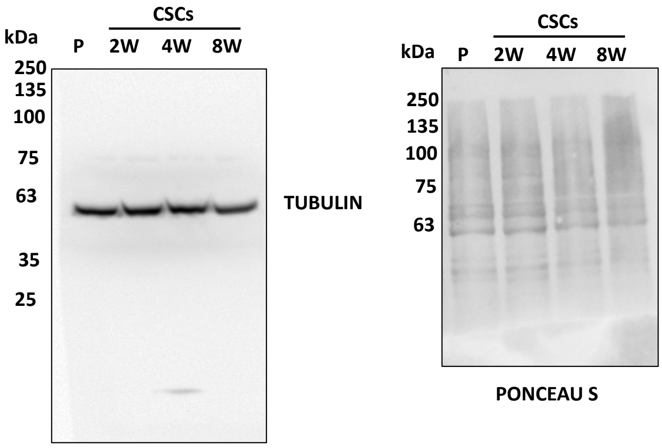

Supplementary blot 13. Full-length western blot from figure 6C. Representative immunoblot of loading controls tubulin and Ponceau S in Panc1 parental (P) cells and CSCs at 2 weeks (2W), 4 weeks (4W), and 8 weeks (8W) of culture. 12% SDS-PAGE gel.
