# Supplementary figures and images for: Mitochondrial Elongation and OPA1 Play Crucial Roles during the Stemness Acquisition Process in Pancreatic Ductal Adenocarcinoma
Source: Cancers (Basel). 2022 Jul 14;14(14):3432. doi: 10.3390/cancers14143432 (PMC9322438; doi:10.3390/cancers14143432)

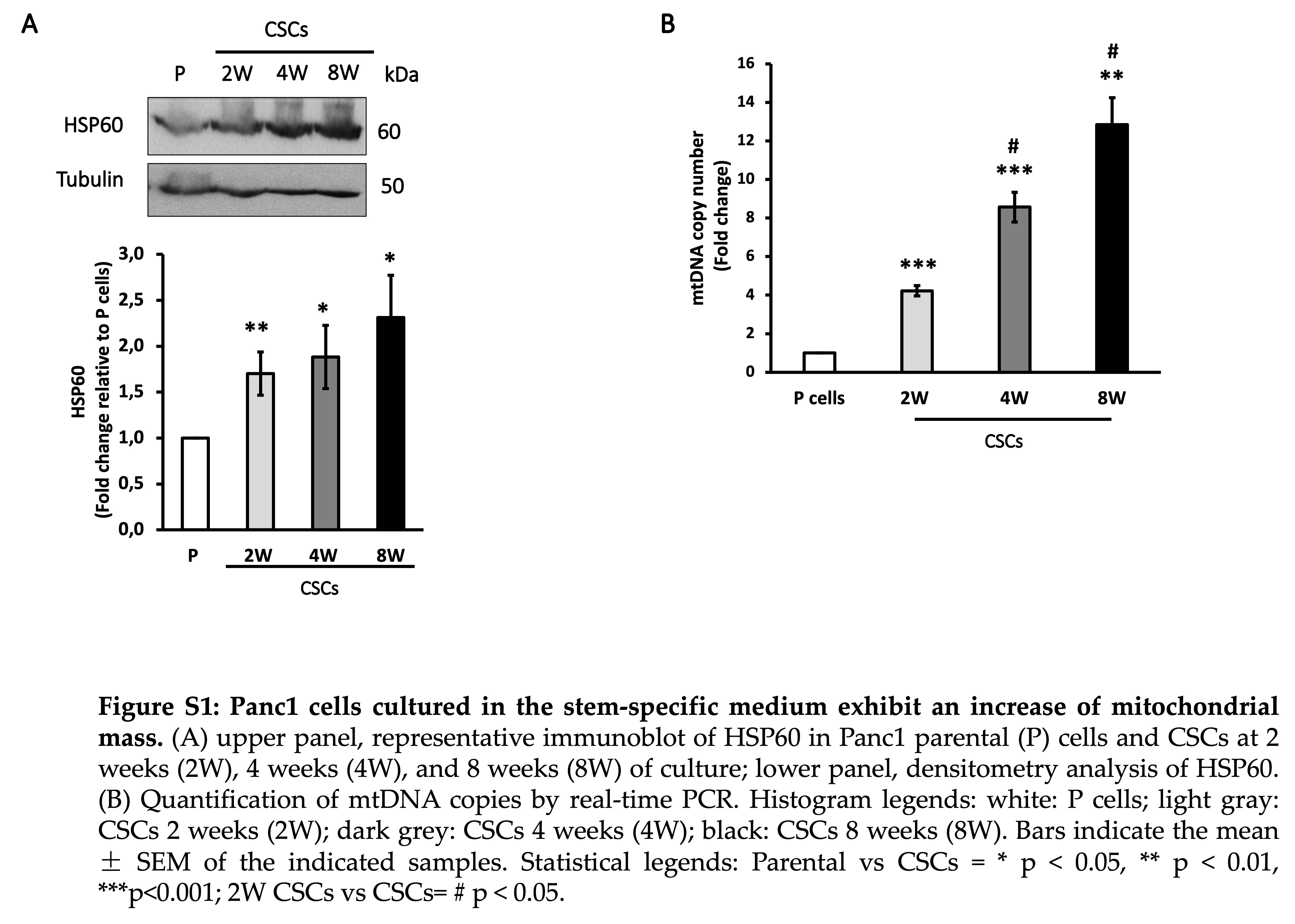

Supplement: Supplementary file 1 [file cancers-14-03432-s001.zip › cancers-1773998-supplementary/Figure S1.jpg]

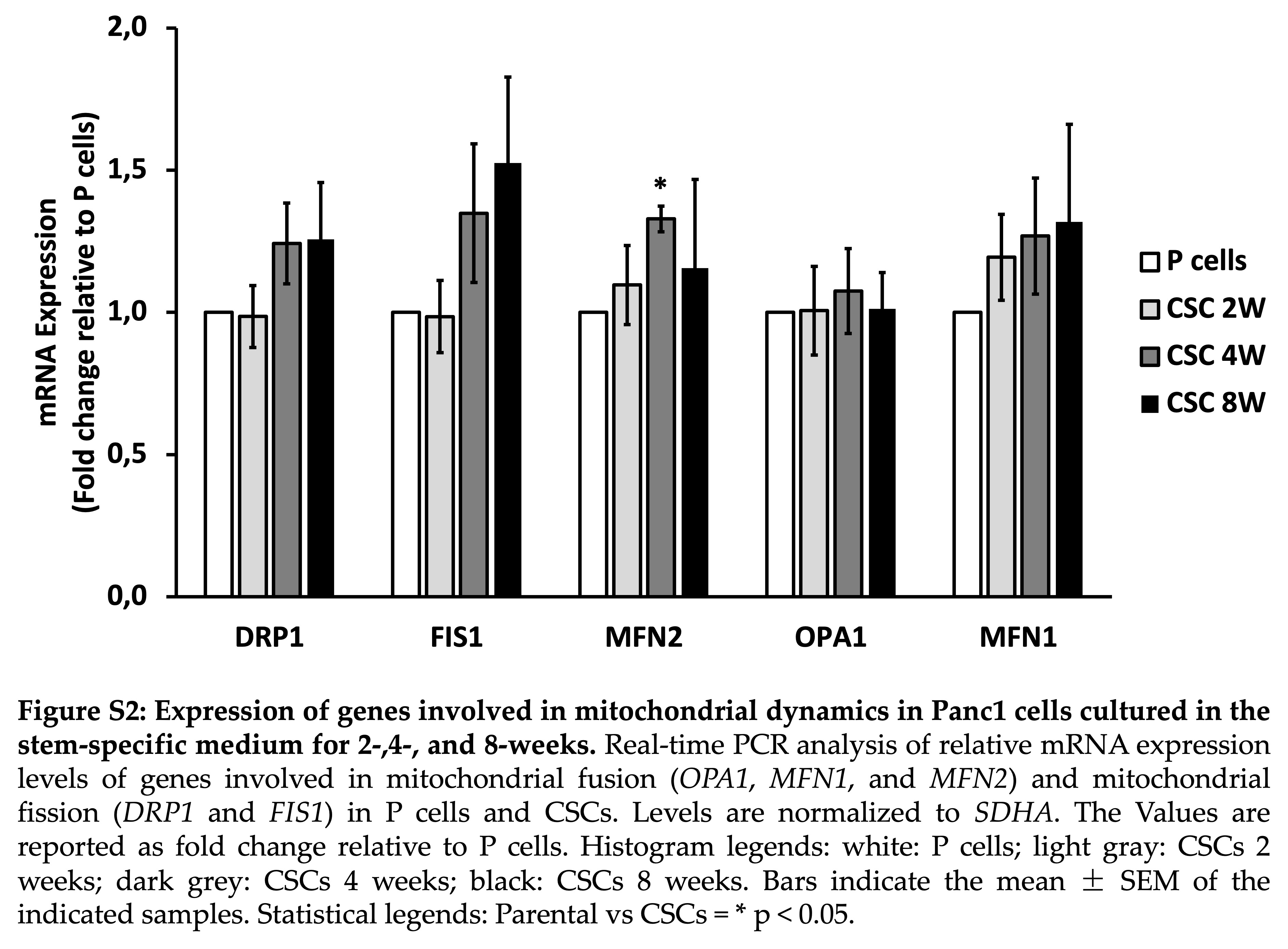

Supplement: Supplementary file 1 [file cancers-14-03432-s001.zip › cancers-1773998-supplementary/Figure S2.jpg]

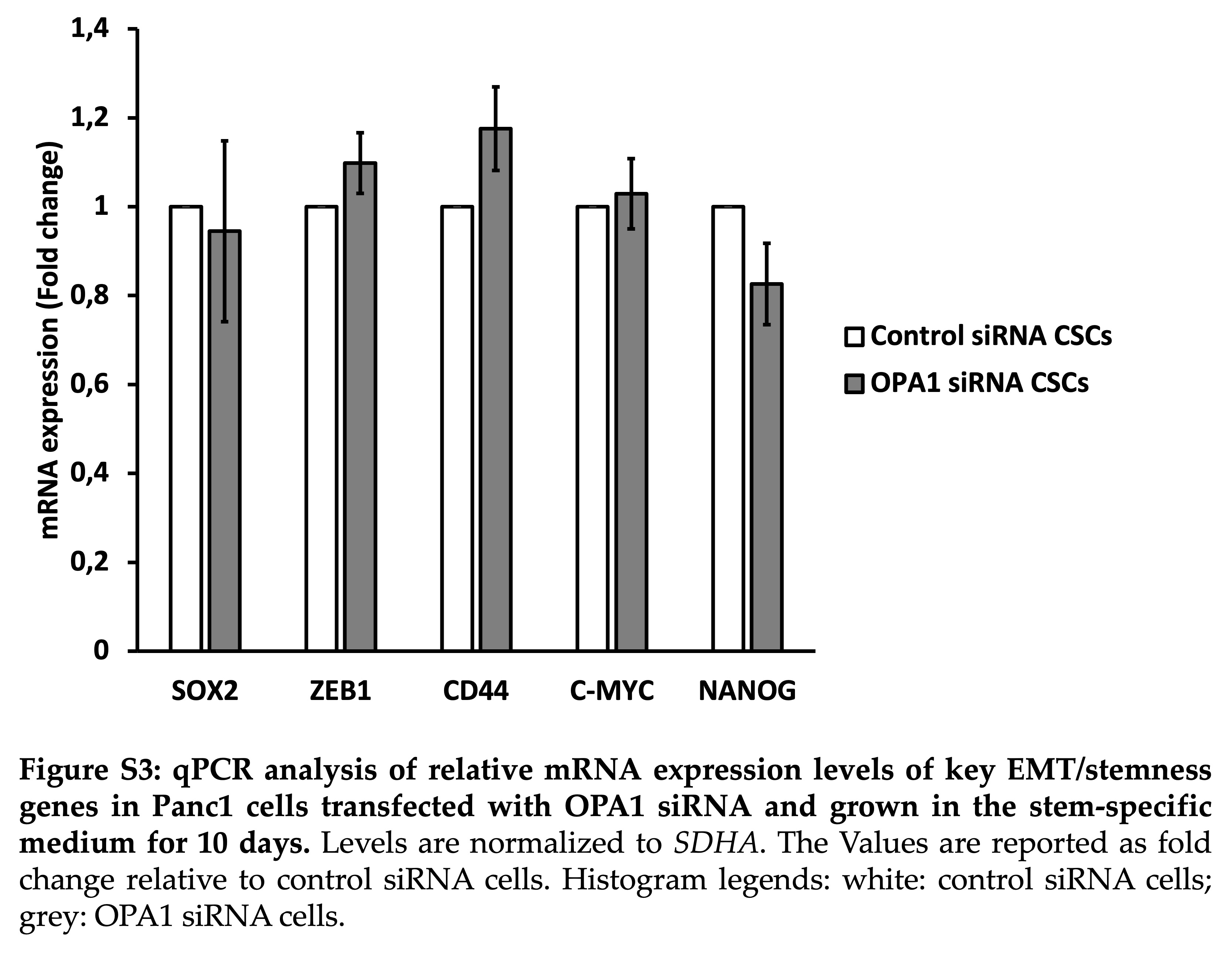

Supplement: Supplementary file 1 [file cancers-14-03432-s001.zip › cancers-1773998-supplementary/Figure S3.jpg]

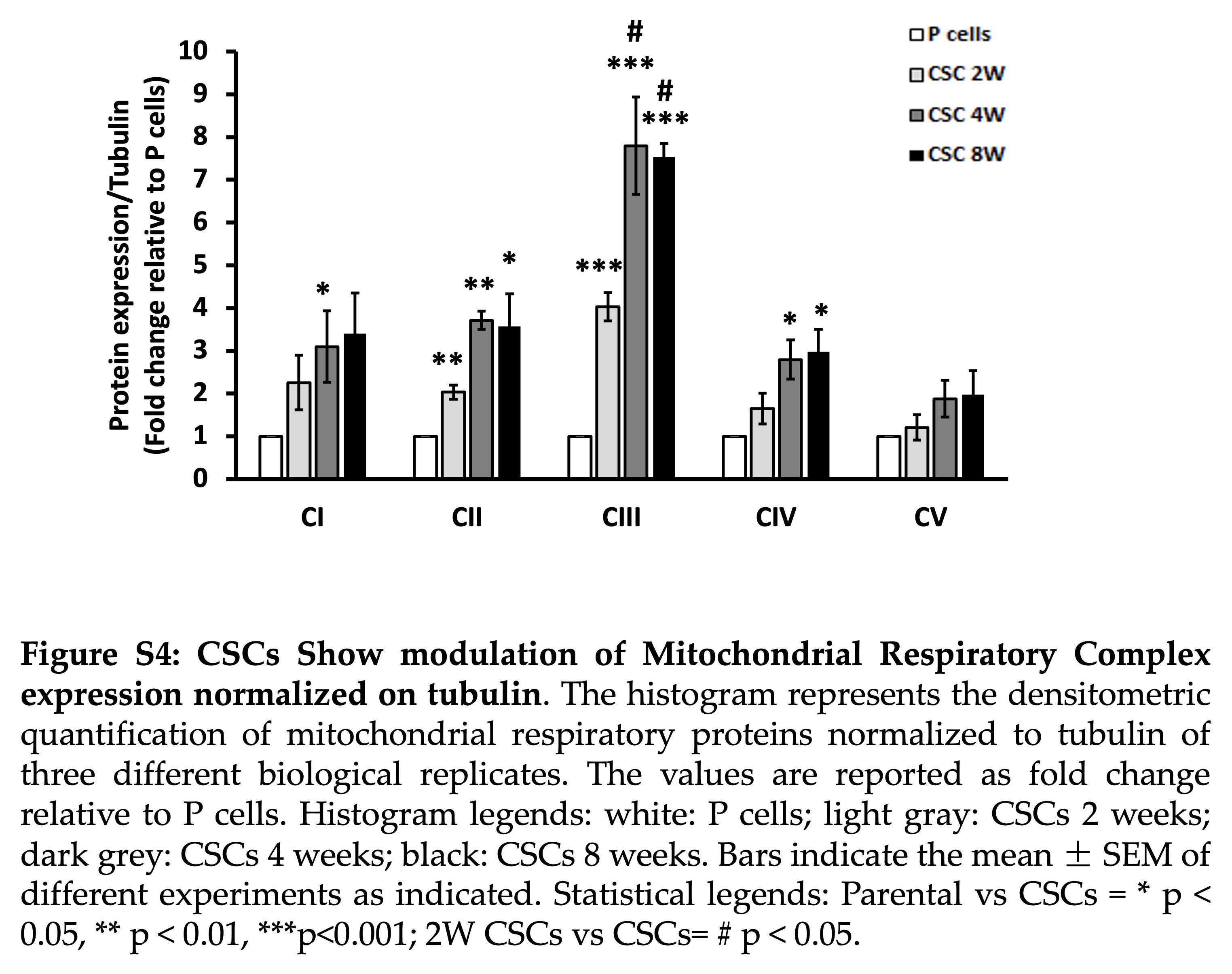

Supplement: Supplementary file 1 [file cancers-14-03432-s001.zip › cancers-1773998-supplementary/Figure S4.jpg]
